# Cleansing efficacy of an auto-cleaning toothbrushing device with nylon bristles: a randomized-controlled pilot study

**DOI:** 10.1007/s00784-022-04755-9

**Published:** 2022-10-22

**Authors:** Mathias Keller, Gregor Keller, Thomas Eller, Lukas Sigwart, Vera Wiesmüller, René Steiner, Vincent Offermanns, Ines Kapferer-Seebacher

**Affiliations:** 1 Department of Operative and Prosthetic Dentistry, Medical University of Innsbruck, Anichstr. 35, 6020 Innsbruck, Austria; 2 Private Practice , Die Kieferchirurgen”, Austraße 51, 6122 Fritzens, Austria

**Keywords:** 10-s toothbrush, Dental biofilm(s), Electric, Oral hygiene, Plaque index

## Abstract

**Objectives:**

To compare the cleansing efficacy of an auto-cleaning device with nylon bristles (*Y*-*brush®*) to that of manual toothbrushing.

**Materials and methods:**

Twenty probands refrained from oral hygiene for 3 days. Rustogi Modified Navy Plaque Index was assessed before and after (randomized) toothbrushing either with the auto-cleaning device for 5 s per jaw or with a manual toothbrush for a freely chosen time up to 4 min. The clinical investigation was repeated in a cross-over design. In a third trial period, the brushing time for auto-cleaning was increased to 15 s per jaw. The study was supplemented by plaster cast analyses.

**Results:**

Full-mouth plaque reduction was higher with manual toothbrushing than with auto-cleaning for 5 s per jaw (*p* < 0.001). There was no statistically significant difference on smooth tooth surfaces but on marginal and interdental sites. Increasing the brushing time of auto-cleaning to 15 s per jaw resulted in a comparable full-mouth plaque reduction as with manual toothbrushing (*p* = 0.177). In 95% of individuals, the device was too short not completely covering second molars. In 30.67% of teeth, the gingival margin was not covered by bristles.

**Conclusions:**

Auto-cleaning devices with nylon bristles have a future potential to reach plaque reduction levels comparable to manual toothbrushing, although manufacturers must focus on improving an accurate fit.

**Clinical relevance:**

Under the premise of an ameliorated fit, the auto-cleaning device might be recommendable for people with low brushing efficacy. Interdental sites remain a failure point if adjunct interdental cleaning is not viable.

## Introduction

**Fig. 1:**
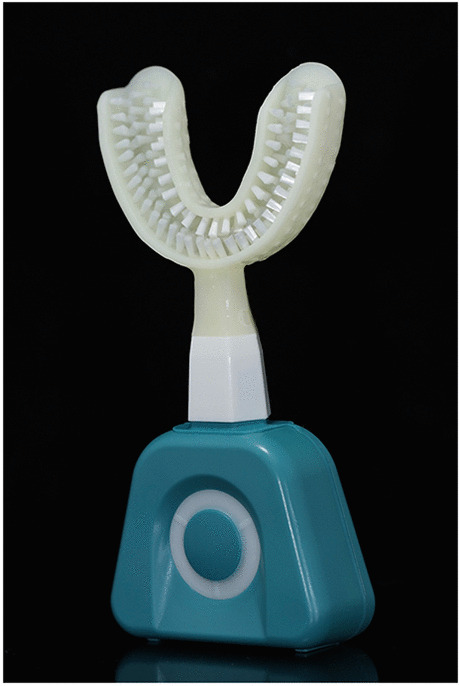
The auto-cleaning device *Y-brush*®. The single-sided and flexible mouthpiece is mounted with six rows of nylon bristles at the occlusal, oral, and vestibular side of the jaw. Bristles are aligned in 45° against the tooth surfaces to simulate the bass method. It must be rotated to the left and right during the cleaning process additional to gently and quickly chewing on it [15]

Dental caries and periodontitis are biofilm-based behavior-mediated diseases, which could be largely prevented by relatively inexpensive measures such as home-based mechanical plaque removal, fluoride application, repeated individualized oral hygiene instructions with professional tooth cleaning, and risk factor control [1–3]. Even in the light of latest insights into the composition and dynamics of the oral microbiome and novel approaches to the management of microbial dysbiosis, domestic oral hygiene remains the cornerstone in the prevention of dental and periodontal diseases [4–8]. Twice daily toothbrushing for at least 2 min with fluoridated dentifrice and the additional use of interdental cleaning devices is universally recommended [9]. Although it is acknowledged how oral health care should be performed, epidemiologic surveys point at a lack of efficient biofilm removal and awareness in the general population. A recent study showed that even after performing oral hygiene to the best of one’s abilities, gingival margins showed persistent plaque at 69.48% ± 12.31% sites (mean ± SD) [10]. Technical developments such as electric toothbrushes have the possibility to improve the efficacy of oral hygiene measures. There is a certainty for a small but statistically significant effect of powered toothbrushes over manual toothbrushes for dental plaque removal [11]. Systematic reviews reported on a weighted mean plaque score reduction of 53% for manual toothbrushing, and 65% plaque reduction was observed when using a powered toothbrush (both for experiments using the Navy plaque index as in the present study) [12, 13]. In addition to the toothbrush used, the effect of toothbrushing depends on patients’ motivation, understanding, and manual dexterity. A fully automated toothbrushing device could exclude most of these factors. The auto-cleaning devices typically consist of a horseshoe-shaped mouthpiece mounted with several rows of vibrating/moving bristles at the oral and vestibular side of the jaw, cleaning all teeth of a jaw in a single mode of action. In a recent clinical study, we compared the cleansing efficacy of the first contemporary auto-cleaning device that was available on the European market (Amabrush®, Vienna, Austria) with that of uninstructed manual toothbrushing and concluded that the auto-cleaning device was not able to sufficiently remove dental plaque and needs further technical development [14]. Plaster cast analyses assessing the quantity/intimacy of bristle contact to the tooth surfaces during the cleaning procedure showed that the alignment and density of the auto-cleaning device’s bristle rows must be improved, assorted device sizes would be necessary to cover different jaw shapes, and that nylon bristles might be superior to silicon bristles. The French *Y-brush*® (Lyon, France) appears to fulfill some of these parameters. It is the first 10-s auto-cleaning device with nylon bristles, inclined at a 45° angle to the gums mimicking the BASS technique (Fig. 1). The single-sided and flexible mouthpiece is available in two different sizes and must be moved to the left and right side during the cleaning process additional to gently and quickly chewing on it [15].

The purpose of this randomized-controlled and single-blinded cross-over study was to compare the cleansing efficacy of an auto-cleaning device with nylon bristles and flexible mouthpiece with that of uninstructed manual toothbrushing. The null hypothesis was that there would be no difference in plaque reduction between the two brushing methods in randomly selected probands.

## Material and methods

The Ethics committee of the Medical University of Innsbruck, Austria, approved the study (ID AN 5123). The study was conducted in accordance with the 1964 Helsinki declaration and its later amendments. All subjects signed an informed written consent prior to the study enrollment.

### Study subjects

Twenty volunteers were recruited from the Department of Operative and Prosthetic Dentistry, Medical University of Innsbruck (Austria). Inclusion criteria were (1) age ≥ 18 years, (2) contractual capability, and (3) the presence of ≥ 5 teeth per quadrant. Exclusion criteria were (1) dental students or professionals, (2) oral hygiene instructions prior to the study, (3) community periodontal index of treatment needs (CPITN) grade 3 or 4 [16], (4) pregnancy or breastfeeding, (5) systemic diseases or conditions that are associated with an increased risk of infection or necessitate concomitant antibiotic therapy with dental treatment, and (6) mental and behavioral disorders that impede (verbal) communication. Recruitment was performed from January 25th to April 2nd 2021, and data collection was carried out at the Department of Operative and Prosthetic Dentistry, Medical University of Innsbruck (Austria) from April 9th to May 14th 2021.

### Clinical intervention

The cleansing efficacy of brushing with the auto-cleaning device *Y-brush*® versus manual toothbrushing was evaluated in a randomized-controlled, examiner-blinded, three-period cross-over study. At the beginning, each subject was asked to attend four appointments. At day 1, the probands were informed about the study procedure, they signed an informed consent, and inclusion and exclusion criteria were proofed. Alginate impressions of both jaws were taken to obtain stone plaster casts for the evaluation of the size and shape of the dental arches and the investigation of the auto-cleaning device’s fit. After plaque disclosing (*2Tone*, Young, Earth City, Mo, USA), professional tooth cleaning was accomplished with an air-polishing device (*Airflow*® prophylaxis master and *Airflow*® Plus powder; both EMS, Nyon, CH), and, if needed, with sonic scalers and rubber cups with polishing paste (*Cleanic*®, Kerr, Bioggo, CH). Each proband was instructed in the handling of the *Y-brush®* (size M, the currently only available size for adults) according to the manufacturer. Then, each proband was instructed to refrain from oral hygiene, including toothbrushing, the use of dental floss or other interdental cleaning devices, and the use of mouth rinses or chewing gum for 3 days. According to a computer-generated randomization (*Microsoft*® Office Excel), probands were allocated either to group 1, designated to start with using the *Y-brush*®, or group 2, determined to start with manual toothbrushing. After 3 days of undisturbed biofilm accumulation, plaque was disclosed and scored by one blinded investigator (MK) using the Rustogi Modified Navy Plaque Index (RMPN) [17] before and after brushing with the assigned device. Probands of group 1 were assisted with using the *Y-brush*® according to the manufacturer’s instructions. The mouthpiece was wetted and loaded with toothpaste (Sensodyne proschmelz, GlaxoSmithKline Pharma GmbH, Vienna, Austria) by use of the supplied silicone toothpaste adaptor. After insertion of the mouthpiece and adjustment to the upper jaw to ensure maximum fit, the start button was pressed. After 5 s (during which the predefined jaw movements were exercised), the brushing automatically stopped. The same procedure was repeated in the lower jaw. The RMNPI was assessed, and teeth were air polished. Probands of group 2 were told to brush their teeth with a manual toothbrush (*Oral B Indicator Medium 35*®, Procter & Gamble UK, Weybridge, Surrey, UK) loaded with the same toothpaste. Tooth brushing was performed without instruction and—in a departure from convention and doctrine—in the absence of a mirror to ensure that the probands had no visual control of the disclosed plaque. The respective manual brushing method was recorded, and the brushing time was chosen freely up to a maximum of 4 min and registered. After rinsing with water, the RMNPI was assessed, and air-polishing was performed. After a wash out phase of 10 days when the probands were practicing their usual oral hygiene procedures, they presented for the third visit. Again, plaque was disclosed, and teeth were cleaned by air-polishing. After abolishing oral hygiene for 3 days, the fourth visit unfolded in analogy to the second visit, with group 1 using the manual brush and group 2 using the auto-cleaning device.

Due to unsatisfactory plaque removal with the used *Y-brush*® mode, we decided to perform a third brushing period. Ten of the initially twenty volunteers agreed to participate, and after a wash out phase of 14 days, they presented for the fifth visit. Plaque was disclosed, teeth were cleaned by air-polishing, and after abolishing oral hygiene for 3 days, the sixth visit unfolded in analogy to the second visit, with all probands using the auto-cleaning device now for 15 s per jaw.

### Rustogi Modified Navy Plaque Index [17]

The index divides buccal and lingual surfaces into nine areas (A to I) that are scored for the presence (score = 1) or absence (score = 0) of plaque. It is based on a dichotomous principle and assesses the presence of plaque on a whole mouth basis (areas A–I), interdental basis (areas D and F), and the gingival margin basis (areas A–C) and thus allows to evaluate each area separately. Third molars and carious teeth were excluded from the evaluation, whereas teeth with fillings, inlays, onlays, or crowns were included. RMPNI is calculated as percentage of biofilm adhering sites to measured sites.

### Statistical analysis

On a proband level, RMNPI values were calculated as the total number of tooth areas with plaque present divided by the total number of tooth areas scored, and median and range are given. The amounts of plaque reduction (pre-minus post-plaque scores) were calculated and mean reduction in the whole mouth, as well as interdental and marginal plaque scores, was compared between the two tooth brushing procedures by Wilcoxon’s signed-rank test. If not stated otherwise, median and range are given. The significance level was set at *p* ≤ 0.01.

## Results

Twenty individuals (10 females and 10 males; all Caucasians) with a median age of 29.3 years (range 20.3–63.1 years) participated in this study. For manual toothbrushing, the median brushing time was 181 s (range 110–240 s).

### Plaque reduction with *Y-Brush®* 5 s per jaw (Table 1)

**Table 1:** Plaque scores before and after cleaning with the *Y-brush®* or manual toothbrushing. Plaque was scored using the Rustogi Modified Navy Plaque Index before (baseline) and after plaque removal. The index divides buccal and lingual surfaces into nine areas that are scored for the presence (score = 1) or absence (score = 0) of plaque. Plaque scores were calculated as the total number of tooth areas with plaque present divided by the total number of tooth areas scored. Plaque reduction was calculated as pre-minus post-cleaning plaque scores. All data are given with median (range). For all (subgroup) analyses, plaques scores were statistically significantly lower after cleaning than before cleaning (*p* < 0.001; not further specified in the table)

	Whole mouth plaque scores (%)		
	Baseline	After cleaning	Plaque reduction
Manual toothbrush	51.4 (23–66.5)	12.4 (7.1–23.4)	37.7 (13.8–53.2)
Y-brush*®* 5 s	51.3 (22.2–71.4)	29.6 (10.1–48.8)	24 (10.5–40.9)
*p*-value	0.110	0.0001	0.0001
	Plaque scores on smooth tooth surfaces I, H, G, E (%)		
	Baseline	After cleaning	Plaque reduction
Manual toothbrush	14.0 (0.5–36.7)	0.5 (0–4.9)	12.9 (0.5–31.7)
Y-brush*®* 5 s	14.3 (4.2–43.8)	3.4 (0–9.4)	9.8 (3.6–39.7)
*p*-value	0.080	0.0004	0.631
	Marginal plaque scores (%)		
	Baseline	After cleaning	Plaque reduction
Manual toothbrush	88.6 (65.4–98.2)	24.9 (5.1–42.6)	60.8 (36.1–86.9)
Y-brush*®* 5 s	86.3 (53.6–100)	51.3 (26.8–83.3)	32.7 (14.2–49.4)
*p***-**value	0.509	0.0001	0.0001
	Interdentale plaque scores (%)		
	Baseline	After cleaning	Plaque reduction
Manual toothbrush	71.4 (4.6–100)	16.5 (2.8–40.2)	43.1 (1.9–72.3)
Y-brush*®* 5 s	78.6 (9.8–99.1)	39.3 (2.8–86.6)	26.4 (4.5–47.3)
*p*-value	0.110	0.0005	0.008

*sec*, seconds

**Fig. 2:**
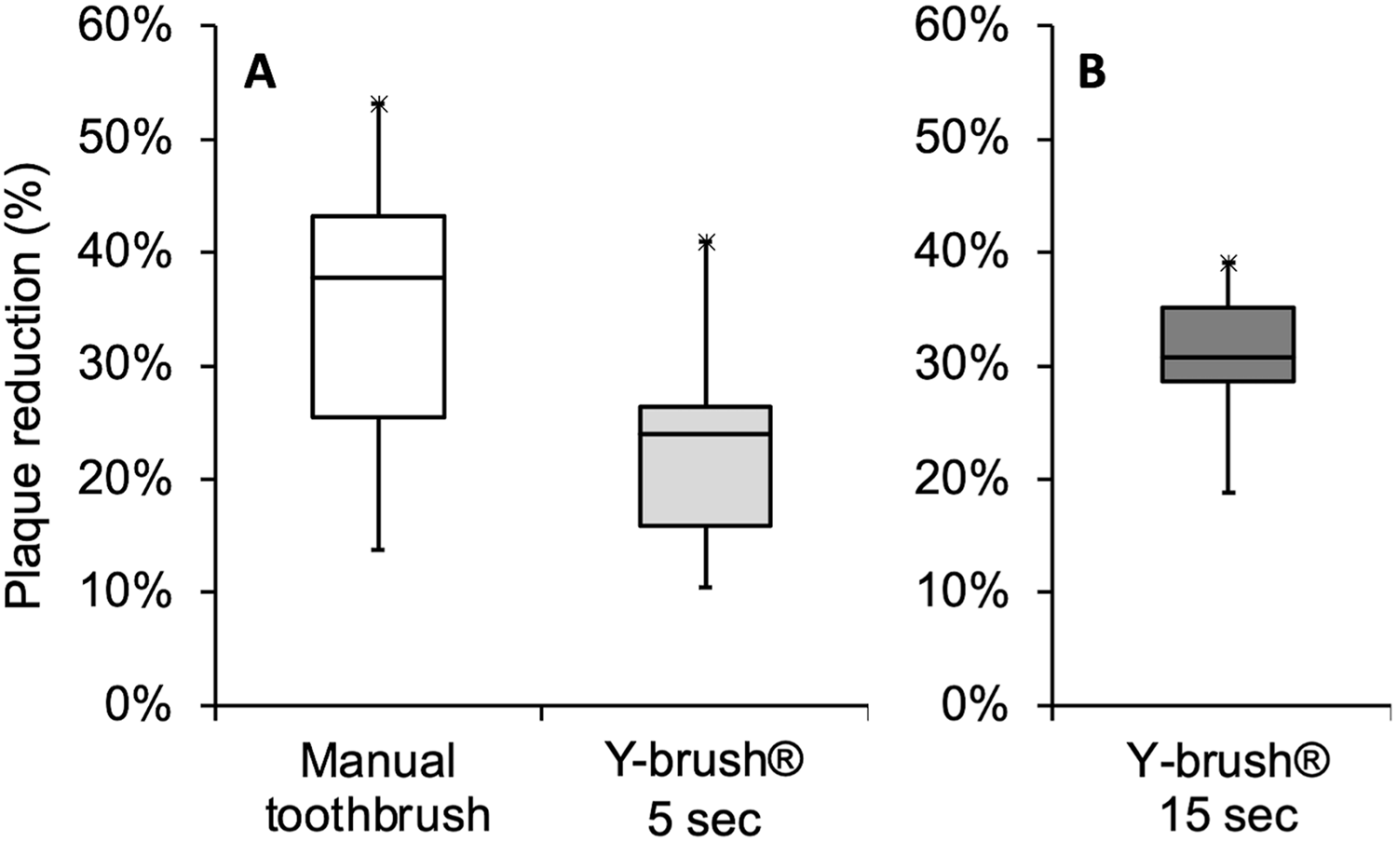
Plaque reduction of whole mouth Rustogi Modified Navy Plaque Index (RMNPI). The RMNPI divides buccal and lingual surfaces into nine areas that are scored for the presence (score = 1) or absence (score = 0) of plaque. On a proband level, RMNPI was calculated as the total number of tooth areas with plaque present divided by the total number of tooth areas scored. The amount of plaque reduction was calculated by subtracting post- from pre-brushing levels. **A** Plaque reduction was statistically higher with manual toothbrushing for 3 to 4 min than for the auto-cleaning device brushing for 5 s per jaw (*p* < 0.001). **B** Ten of the initially twenty volunteers agreed to participate in a third study period, using the auto-cleaning device now for 15 s per jaw, which resulted in a non-statistically significant difference compared to manual brushing (*p* = 0.177)

After 3 days of plaque accumulation, full-mouth RMNPI was 51.39% (range 23.05–66.47%) for the investigation of manual toothbrushing and 51.34% (22.22–71.43%) for the auto-cleaning device (*p* = 0.109). Subgroup analyses of anterior and posterior teeth as well as buccal and lingual dental surfaces and marginal and interdental sites revealed no statistically significant differences between baseline plaque scores (*p* > 0.05). Immediately after brushing, statistically significant reductions in whole mouth plaque scores were observed for manual toothbrushing with a median of 37.70% (13.79–53.17%; *p* < 0.0001) as well as for the auto-cleaning device with a median of 24.01% (10.49–40.87%; *p* < 0.0001) (Table 1). Reduction of full-mouth RMNPI was statistically significantly lower and post-brushing plaque indices were statistically significantly higher after brushing with the auto-cleaning device than with manual toothbrushing (*p* < 0.0001) (Fig. 2).

**Fig. 3:**
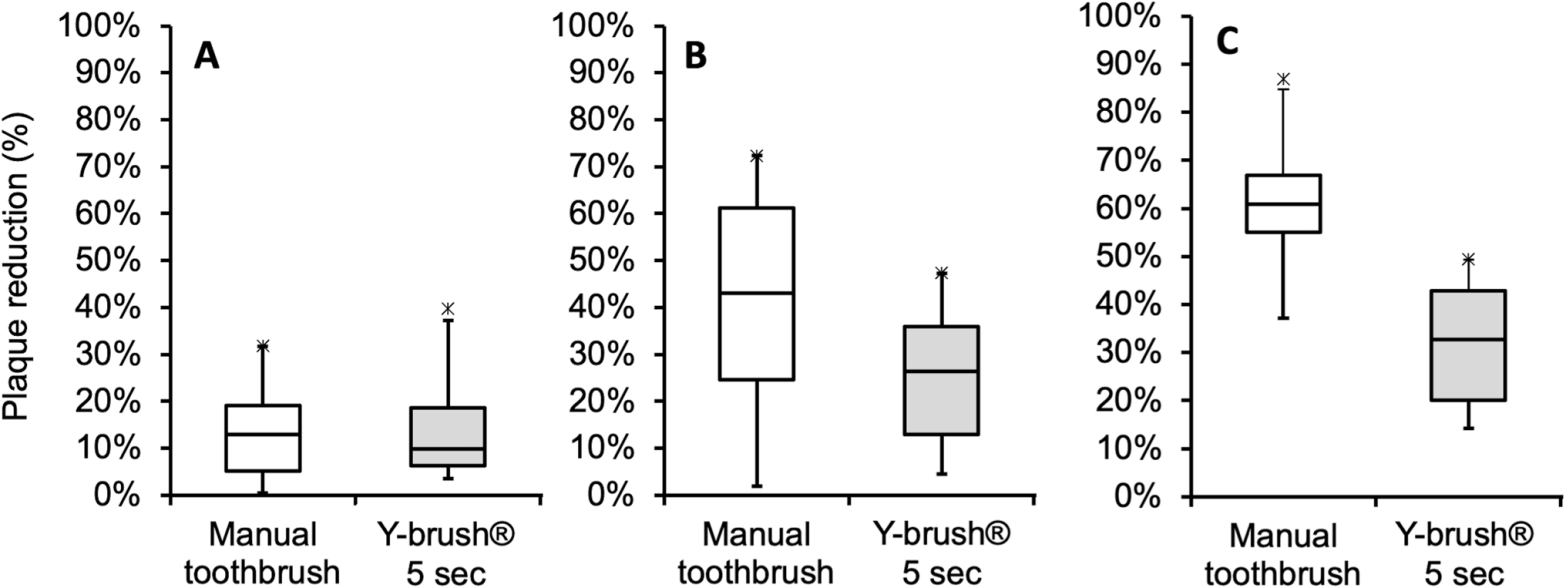
Subgroup analysis of plaque reduction. **A** Smooth tooth surfaces. After brushing with the auto-cleaning device for 5 s per jaw, smooth tooth surfaces showed no statistically significant difference for plaque reduction compared to manual toothbrushing. **B** Interdental and **C** marginal areas showed statistically significantly lower plaque reduction after brushing for 10 s per jaw with the auto-cleaning device compared to manual toothbrushing (*p* < 0.01)

Subgroup analyses (see Table 1) for buccal, lingual, smooth, marginal, and interdental areas revealed statistically significant reductions of plaque scores for all areas with the manual toothbrush as well as the auto-cleaning device (*p* < 0.0001). On smooth tooth surfaces I, G, H, and E, there was no statistically significant difference of plaque reduction between manual toothbrushing (median 12.92%; range 0.47–31.74%) and the auto-cleaning device (9.82%; range 3.57–39.73%) (*p* = 0.627). In contrast, marginal areas A, B, and C (32.74%; range 14.20–49.36% and 60.81%; range 36.31–86.90, respectively) and interdental areas F and D (26.37%; range 4.46– 47.32% and 43.10%; range 1.85–72.32%, respectively) showed statistically significantly lower plaque reduction with the auto-cleaning device compared to manual toothbrushing (*p* < 0.01) (Fig. 3).

### Plaque reduction with *Y-Brush®* 15 s per jaw

For evaluating the effect of longer brushing with the auto-cleaning device, we increased the brushing time per jaw from 5 to 15 s. Ten probands were willing to participate in this second part of the study (non-blinded, non-randomized). After 3 days of plaque accumulation, there was no statistically significant difference for pre-brushing RMNPI compared to the first part of the trial (median 59.65%; range 18.52–71.23%; *p* > 0.05). Immediately after brushing with the *Y-brush®* for 15 s per jaw, statistically significant reductions in whole mouth plaque scores were observed (median reduction 30.73%; range 8.44–39.09%; *p* = 0.005) which were statistically significantly higher than for the 5-s brushing mode (*p* = 0.007) and not statistically significantly different from plaque reduction with manual toothbrushing (*p* = 0.177). Subgroup analyses revealed still lower plaque reduction on marginal areas with a tendency to statistical significance (manual toothbrushing 58.88%, range 36.31–86.9%, and *Y-Brush®* 15 s 41.2%, range 17.9–67.9%, respectively) (*p* = 0.01) (Table 1). On smooth tooth surfaces, the auto-cleaning device reached non-statistically significantly higher plaque reduction than manual toothbrushing (22.12%, range 13.84–31.25% versus 8.38%, range 0.47–31.74%, respectively, *p* = 0.09), although post-brushing overall plaque scores were still statistically significantly higher for the *Y-brush®* 15-s mode (*p* < 0.01).

### Plaster cast analysis

**Fig. 4:**
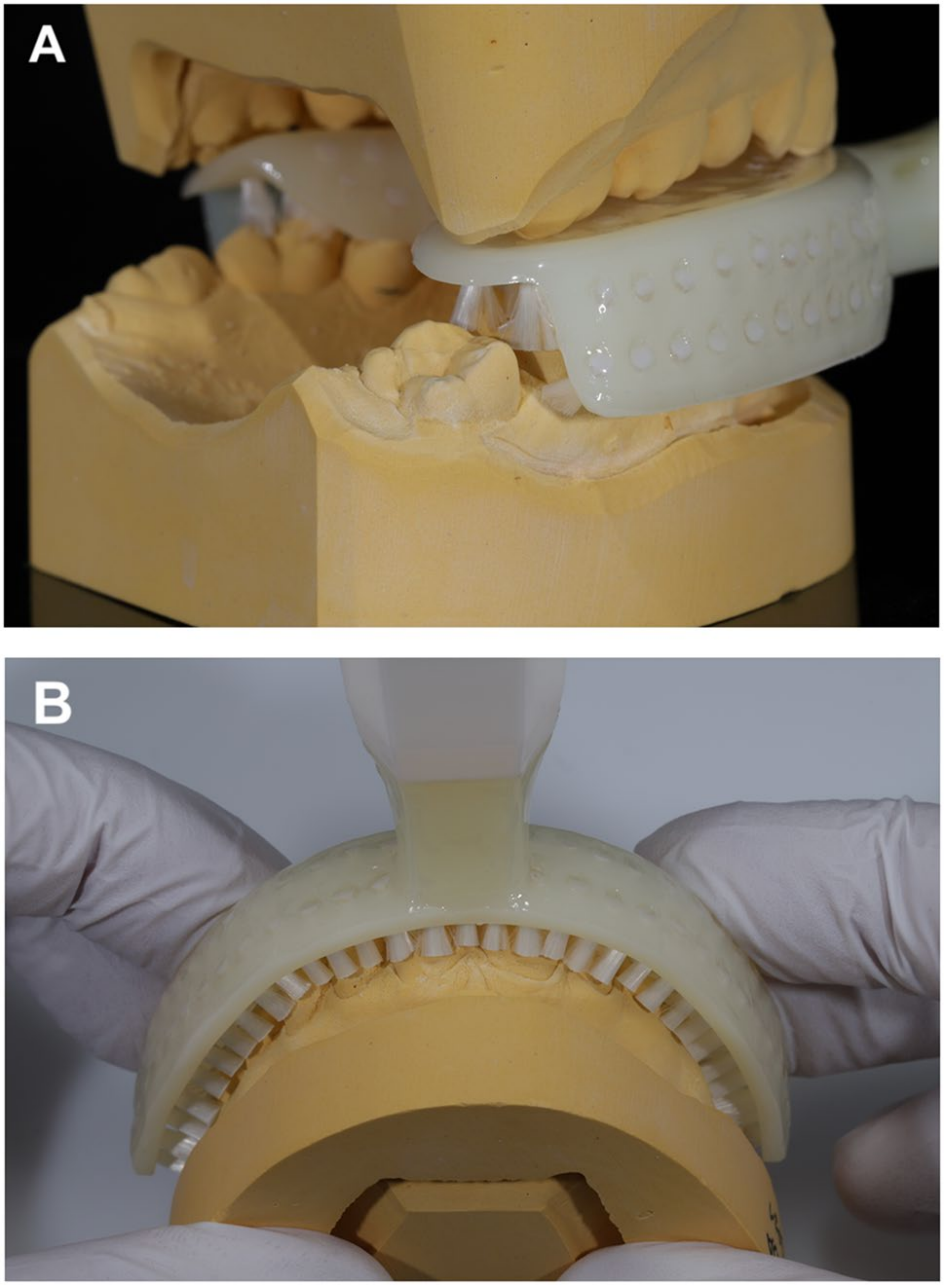
Assessment of accurate fit on plaster casts. **A** In all but one individual, the mouthpieces were too short not completely covering the second molars in the lower and/or upper jaw (range 2–11 mm) resulting in increased plaque retention. **B** In 382 (30.67%) teeth, the gingival margin was not covered by bristles when pushing the mouthpiece gentle on the plaster cast (mean distance 3.13 ± 2.30 mm; range 0.5–9 mm). Number of plaque-positive marginal areas was statistically significantly higher in teeth not covered by the mouthpiece than in marginal areas covered by the bristles (1.81 ± 1.16 and 1.59 ± 1.23, respectively; *p* = 0.009)

Brushing efficacy of the auto-cleaning device was further analyzed regarding the widths and lengths of the jaw arches. No statistically significant correlations were found between the widths of the jaws and post-brushing plaque indices. There were statistically significant correlations between the post-brushing plaque indices and the length of the upper and lower jaw with *r* = 0.64 (95% CI 0.27–0.84; *p* = 0.003) and *r* = 0.45 (95% CI 0.10–0.74; *p* = 0.045), respectively. We then investigated the accurate fit of the mouthpieces on the plaster casts. In all but one individual, the mouthpieces were too short, not (completely) covering the second molars in the lower and/or the upper jaw (Fig. 4) which correlated with plaque retention on the respective teeth. In total, 62-second molars were not fully covered by the mouthpiece with a mean of 5.58 ± 3.46 mm (range 2–11 mm). Pearson’s correlation coefficient for post-brushing plaque retention on second molars (= number of areas of the appropriate teeth with plaque) and the distance not covered by the mouthpiece (measured on the occlusal surface) was *r* = 0.36 (95% CI 0.12; 0.59; *p* = 0.001).

Then, we looked in detail on the marginal areas as on these sites the plaque reduction was unsatisfactory even after brushing with the 15-s mode of the auto-cleaning device. In 382 teeth (30.67%), the gingival margin was not covered by the bristles when pushing the mouthpiece gently on the plaster cast. For these teeth, the distance between the bristles and the gingival margin was 3.13 ± 2.30 mm (range 0.5–9 mm). Number of plaque-positive marginal areas (measured during the clinical trial) was statistically significantly higher in teeth marginally not covered by the mouthpiece than in marginal areas covered by the bristles in plaster cast analyses (1.81 ± 1.16 and 1.59 ± 1.23, respectively; *p* = 0.009).

## Discussion

Tooth brushing efficacy is largely dependent on patients’ motivation, manual dexterity, and knowledge of how to brush. It was recently shown that the efficacy of toothbrushing is not a matter of brushing time but of establishing brushing systematics leading to evenness of distribution of brushing time [18]. In contrast, brushing behaviors of laypeople are characterized by uneven distribution of brushing time resulting in a neglect of palatinal surfaces and by a large portion of scrubbing [18–20]. Auto-cleaning devices could not only overcome these limitations but could also increase the compliance by promising “clean teeth within ten seconds” which seems indeed tempting. Although when following the advice of brushing with a manual or electric toothbrush for at least 2 min [21], a fully dentate individual brushes each tooth only for a mean of 4 s.

In a recent study investigating an auto-cleaning device with silicon nubs (*Amabrush®*, Vienna, Austria), we concluded that this innovative approach is still in its infancy and in need for substantial improvement [14]. None of the individuals reached an equal or higher plaque reduction with that auto-cleaning device (range of plaque reduction 6 to 19%) compared to manual toothbrushing (range 22 to 44%). In subgroup analyses, there was one area where the auto-cleaning device was equally efficient in plaque reduction as the manual toothbrush. Mean plaque reduction on the palatal aspects of upper molars and premolars was 12.18 ± 6.96% for the auto-cleaning device and 13.55 ± 8.63% for the manual toothbrush (*p* = 0.586) [14]. Thus, lack of a statistically significant difference in this area was not due to a higher brushing efficacy of the auto-cleaning device but due to lower plaque reduction with the manual toothbrush compared to other regions. The authors recommended a (re)evaluation of the bristle alignment towards the tooth surfaces. The bristles should be made of nylon to pack them more densely. Another problem was the large width of the mouthpiece which might have adjoined to the ascending branch of the mandible and, thus, was deviated forwardly. The French *Y-brush®* seems to fulfill some of the recommended criteria; therefore, it was chosen for the present investigation. Indeed, mean of whole mouth plaque reduction with the *Amabrush®* was 11.37 ± 3.70% compared to the *Y-brush®* with 22.60 ± 8.06%, which was additionally increased to 29.95 ± 8.42% by a longer brushing mode of 15 s per jaw. It is worth to mention that there were no differences in plaque scores of the control intervention manual toothbrushing between the clinical trials of Schnabl et al. (2021) (31.39 ± 5.27%) and the present one (35.36 ± 10.48%).

In the present study, we decided to assess the Rustogi Modified Navy Plaque Index [17], a dichotomous index evaluating plaque presence or absence in nine areas on buccal and lingual surfaces which is a quite time-consuming procedure. It allows to assess plaque levels on a full-mouth level, but also subgroup analyses for smooth surfaces, interdental and gingival margin areas separately. A disadvantage of dichotomous plaque indices is the variability of plaque amounts which requires intense examiner calibration in the case of multiple investigators or—as in our study—one trained investigator measuring all plaque indices. The authors’ main arguments to use dichotomous plaque indices are that statistical analyses of ordinal indices are difficult to translate to daily clinic, and secondly, the most frequent way to analyze ordinal plaque scores is to treat them as metric variables, calculating mean ± standard deviation or median and interquartile range of all measured sites and using non-/parametric statistical tests, which is questionable from a statistical point of view [22–24].

In the authors’ opinion, the development of auto-cleaning devices seems a gratifying approach to increase both the frequency and the efficacy of toothbrushing which have been ascertained to be insufficient in the majority of adults, adolescents, and children [25–27]. Therefore, we undertook analyses to spot the reasons for the lack of efficacy of the *Y-brush*® design. Improper intimacy of bristle contact to the tooth surfaces should be no limitation of the *Y-brush®* as the mouthpiece is bendable and is moved to the left and right during the brushing procedure. This hypothesis is supported by plaster cast analyses and the fact that smooth tooth surfaces were sufficiently cleaned with the auto-cleaning device with post-brushing plaque levels of 3.4% (range 0–9.4%). Insufficient cleaning was evident after 5 s of brushing per jaw on marginal surfaces with post-brushing plaque levels of 51.5% (26.8–83.3%) and interdental areas with post-brushing plaque levels of 39.3% (2.8–86.6%). A longer brushing mode increased plaque reduction also in marginal areas, although it was still lower compared to manual toothbrushing with a tendency to statistical significance (*p* = 0.01). Plaster cast analyses revealed that in 30.67% of investigated teeth marginal areas were not covered by bristles and these sites showed statistically significantly higher plaque levels than marginal areas covered by the bristles (*p* = 0.009). To overcome this limitation, the manufacturer instructs the users in their videos to bite on the mouthpiece gently and quickly during the brushing procedure. The distance between the apical bristle row and the gingival margin was in mean 3.13 ± 2.30 mm (range 0.5–9 mm) which seems to be too much to be compensated by occlusal pressure. An additional bristle row might increase the brushing efficacy in marginal areas.

Additional plaster cast analyses revealed that for all but one individual, the mouthpiece was too short consequently not completely covering second molars with a range of 0.5 to 11 mm. The manufacturer recommends moving the mouthpiece gently from one side to the other during the brushing process probably to overcome a non-accurate fit. This movement might compensate some millimeters. In the present study, the distance of the occlusal surface not covered by the mouthpiece in rest was statistically significantly correlated with plaque retention on appropriate teeth (*r* = 0.36; *p* = 0.001). We recommend selecting a mouthpiece that fully or at least nearly covers the occlusal surfaces of all teeth although to date a guide is missing how lay people should select the appropriate size. The width of the jaws does not seem to matter, probably due to the flexible material.

The limitation of the present study is that the elongated brushing mode of the *Y-brush®* was investigated in a non-blinded and non-randomized trial arm and must be reproduced. The brushing time for manual toothbrushing was rather long with 3 to 4 min. Probably, study participants increased the brushing time due to the setting of a clinical trial. Further studies should investigate longer brushing modes and investigate the effect of different mouthpiece sizes in correlation to different jaw lengths and plaque reduction. Furthermore, the number of participants of the present study is rather low, especially for testing a new brush design. Clinical trials with higher numbers of participants and parallel group design testing home use of the auto-cleaning device should be performed. Improvement of the cleaning efficacy of marginal sites is essential, e.g., with a further bristle row.

## Conclusion

This study provides first evidence that auto-cleaning devices may have a future potential to reach plaque reduction levels comparable to manual toothbrushing. The most blatant shortcomings are owed to its insufficient fit resulting in high plaque levels especially at critical marginal and interdental sites. New manufacturing processes may enable the production of individualized mouthpieces which seems to be a prerequisite to adapt the shape of the auto-cleaning devices to the variety of natural dentitions. Future long-term studies are needed.
